# Research on the evaluation method of English textbook readability based on the TextCNN model and its application in teaching design

**DOI:** 10.7717/peerj-cs.1895

**Published:** 2024-02-29

**Authors:** Ying Qin, Azeem Irshad

**Affiliations:** 1 School of Foreign Languages, Wuzhou University, Wuzhou, China; 2 Department of Computer Science and Software Engineering, International Islamic University, Islamabad, Pakistan

**Keywords:** English reading, Deep learning, TextCNN, Readability assessment

## Abstract

English is a world language, and the ability to use English plays an important role in the improvement of college students’ comprehensive quality and career development. However, quite a lot of Chinese college students feel that English learning is difficult; it is difficult to understand the learning materials, and they cannot effectively improve their English ability. This study uses a convolutional neural network to evaluate the readability of English reading materials. It provides students with English reading materials of suitable difficulty based on their English reading ability so as to improve the effect of English learning. Aiming at the high dispersion of students’ English reading level, a text readability evaluation model for English reading textbooks based on deep learning is designed. First, the legibility dataset is constructed based on college English textbooks; second, the TextCNN text legibility evaluation model is constructed; finally, the model training is completed through parameter adjustment and optimization, and the evaluation accuracy rate on the self-built dataset reaches 90%. We use the text readability method based on TextCNN model to conduct experimental teaching, and divided the two groups into comparative experiments. The experimental results showed that the reading level and reading interest of students in the experimental group were significantly improved, which proved that the text readability evaluation method based on deep learning was scientific and effective. In addition, we will further expand the capacity of the English legibility dataset and invite more university classes and students to participate in comparative experiments to improve the generality of the model.

## Introduction

With global integration, the rapid development of economy and technology, and the intensified competition brought about by the entry into the WTO, China has paid more and more attention to English education, and China’s college English education policy system has become increasingly sound, which has greatly improved the quality of higher education. The “English Curriculum Standards for College Students” promulgated by the Chinese Ministry of Education in 2020 divides English language skills into five areas: listening, speaking, reading, reading, and writing. Among them, reading, as a comprehension skill, has a strong comprehension. English reading not only tests the accumulation of language learners’ basic knowledge but also tests the learners’ comprehension ability, generalization ability, logical reasoning ability and evaluation ability. Tercanlioglu (2001) believe that English reading is a skill that people need in daily life and academic life, an important means to improve other language skills, and plays a vital role in grammar and vocabulary learning. English reading is a basic skill that English learners need to master. It is an important way to understand the objective world, develop intelligence and emotion, improve practical ability and thinking ability, and understand the cultures of various countries. It is also an important way to absorb English language information, increase language knowledge, and expand vocabulary. It is an important indicator to measure English ability, so it is very important to improve students’ English reading ability.

There is currently no single standard concept for the definition of reading. Goodman (1967) proposed that English reading is a kind of psychological activity, which is the collision between learners’ internal situational imagination and the language of reading learning materials. Wang (2007) pointed out that reading is the language interaction between the learner and the author, and the learner generates a meaningful interactive learning process between the learner and the author by decoding the encoded information presented by the author. Gorin (2006) defines reading as the silent language and thinking activities of learners. Williams (1984) proposed that reading is the process by which readers see text and understand its meaning. Zawodniak & Kruk (2017) proposed that reading is a process in which readers interact with authors through text content. Swanborn & Glopper (2002) believes that reading is a reader’s ability to use and coordinate multiple methods and skills to process complex information and then understand the content of the text. Tercanlioglu & Demiröz (2015) believes that reading is a complex and multifaceted activity, and readers practice reading by coordinating cognitive, metacognitive, motivational, and social processes. From the viewpoints of the above scholars, it can be seen that English reading is a complex cognitive activity of decoding symbols, a language interaction process between learners and authors, and a process in which learners actively explore and acquire effective information for internalization.

English reading ability refers to the ability to use language knowledge, language ability, reading skills and a certain speed to read English successfully. It is a comprehensive language comprehension skill. Erler (2012) pointed out that reading ability is one of the most important skills in a learning environment, and it is through reading that people learn new information and have the opportunity to obtain other interpretations of certain information. Many researchers have carried out a lot of research on how to improve students’ English reading ability. Rahimi (2009) proposed a strategy for training reading comprehension, pointing out that there is a close relationship between listening comprehension and reading comprehension ( ${\rm r} = 0.90$), and the improvement of one will directly lead to the improvement of the other. Mytkowicz (2014) found that metacognitive reading strategy awareness plays an important role in reading comprehension and education. Yüksel & Yüksel (2012) found that students who used reading strategies performed better on English reading proficiency tests/courses. Wyse & Jones (2005) found that the effective use of reading strategies can help the planning and interactive design of English reading and reading teaching and improve learners’ reading ability. Sheorey & Mokhtari (2001) found that learners with high reading ability and learners with low reading ability have differences in the use of reading strategies.

Krashen (1985) proposed the “input hypothesis,” also known as the “ ${\rm i} + 1$” theory, where “ ${\rm i}$” represents the language learner’s existing language knowledge level, and “ ${\rm i} + 1$” represents the language slightly higher than the learner’s existing language level material, the theory expounds and analyzes the methods and processes that shape language acquisition. According to Krashen’s input hypothesis and the conclusions of many other linguists, the most effective way to improve reading comprehension is to provide students with reading material that is slightly above their reading ability. If the difficulty of the text is too low, students will feel dull or even bored; if the difficulty of the text is too high, students will feel a lack of confidence and interest. Therefore, it is necessary to scientifically evaluate the readability of English reading materials in colleges and universities and match English reading materials with appropriate readability according to students’ reading ability so as to provide English teachers with targeted teaching opinions and to provide learning for parents and students. Suggestions to improve students’ English reading ability and knowledge acquisition efficiency.

Based on the *status quo* of other researchers mentioned above, in order to improve students’ interest in English learning at different levels, and based on the theory of “input hypothesis”, this article proposes the following contributions:

1) This article uses convolutional neural networks to design an English text readability evaluation model to evaluate the readability of college English learning materials;
2) Through a series of data collection and processing processes, this article constructs an English text readability data set for college students to verify the model.

## Related works

American linguist Dale first defined text readability. He believed that text readability is the sum of factors that affect the reader’s understanding of the text, reading speed, and the reader’s interest in the content of the text (Dale & Chall, 1949). At present, the research in academia mainly revolves around traditional feature-based legibility formula evaluation methods and deep learning-based legibility assessment methods.

The Flesch (1948) Reading Ease formula evaluates the text’s scores on a percentile scale. The score value of the evaluation is generally between 0 and 100. The higher the score value, the lower the difficulty of reading the text, and the easier it is for readers to understand correctly. As shown in Eq. (1), ASL can be obtained by dividing the number of words by the number of sentences, which represents the average sentence length. ASW can be obtained by dividing the number of syllables by the number of words, and it represents the average number of syllables in a word. As ASL or ASW increases, the length of sentences or words in the text increases, and therefore, the difficulty of reading the text increases.

(1) $${\rm R}{{\rm G}_{{\rm FRE}}} = 206.835 - 1.015 \times {\rm ASL} - 84.6 \times {\rm ASW}$$

The Gunning (1952) FOG legibility formula evaluates the legibility level of text according to the grade level of American students. For example, a text readability score of 7.0 means that American students in the seventh grade can correctly understand the content of the text; as shown in Eq. (2), ASL can be obtained by dividing the number of words by the number of sentences, which represents the average sentence length. DWR represents the percentage of words marked as difficult words to the total number of words. The formula defines difficult words as words with three or more syllables.

(2) $${\rm R}{{\rm G}_{{\rm GF}}} = 0.4 \times \left( {{\rm ASL} + 100 \times {\rm DWR}} \right)$$

The Automated Readability Index formula also evaluates text readability according to the grade level of American students. As shown in Eq. (3), ACW can be obtained by dividing the total number of letters by the number of words, which represents the average number of letters in a word. ASL is obtained by dividing the number of words by the number of sentences, and it represents the average sentence length.

(3) $${\rm R}{{\rm G}_{{\rm ARI}}} = 4.71 \times {\rm ACW} + 0.5 \times {\rm ASL} - 15.59$$

The Dale-Chall formula (Smith & Senter, 1967) is a vocabulary-based legibility assessment method, as shown in Eq. (4). The method uses a vocabulary of 3,000 words that is familiar to 80 percent of U.S. fourth-graders. This method marks all words that are not in this list as difficult words and then uses the proportion of difficult words to evaluate the difficulty of reading. The Dale-Chall formula combines the average sentence length and the proportion of difficult words to evaluate text readability. where WPS can be obtained by dividing the number of words by the number of sentences, representing the average sentence length. And DWR represents the percentage of words marked as difficult words to the total number of words.

(4) $${\rm R}{{\rm G}_{{\rm DC}}} = 0.1579 \times \left( {{\rm DWR} \times 100} \right) + 0.0496 \times {\rm WPS }$$

However, methods based on legibility formulas make it difficult to capture the deep semantic features of words and cannot represent texts richly, which limits the reading difficulty of evaluating texts. In recent years, with the development of artificial intelligence technology, researchers have begun to explore legibility assessment methods based on deep learning.

In their research, Xia, Kochmar & Briscoe (2016) proposed a graded dataset for the legibility analysis of non-native language learners, The Cambridge Exams dataset (Dale & Chall, 1948), aiming at the legibility of the learning materials of the second language learners, and explored how to solve the problem of how to deal with the lack of sufficient information for the second language learning. In the case of learner data, generalization methods are used to adjust the model based on native language data, and domain adaptation and self-training techniques are explored to improve the system’s ability to process second language learner data. Fujinuma’s experiments show that GCN can achieve high accuracy even with a small amount of labeled data by capturing the difficulty relationship between words and documents (Xia, Kochmar & Briscoe, 2016). Jiang et al. (2020) found that there are significant differences in the performance of the dual-meta model, LSTM, and bidirectional LSTM in the three corpora Newsela, Weebit, and Onestopenglish (Fujinuma & Hagiwara, 2021). Pilán, Volodina & Zesch (2016) found that morphological features also maintained strong predictive power when transferred between L2 input and output texts, and that syntactic and count features were low in informativeness in cross-domain contexts, but were not used for misnormalization of L2 output texts. After the latter, the transfer effect of the latter is better (Jiang et al., 2020). Gonzalez-Garduo & Sgaard (2017) combined a learned model and a linear model to predict the legibility of sentences (Pilán, Volodina & Zesch, 2016). Pilán, Volodina & Zesch (2016) found that, in addition to lexical frequency and inflection, the length of dependencies and the number and type of vocabularies are also valuable for predicting language proficiency (Gonzalez-Garduo & Sgaard, 2017). Stajner, Ponzetto & Stuckenschmidt (2017) established a standard sentence complexity dataset, including original, manually simplified and automatically simplified sentences, and used scores (1–5 points) to evaluate their absolute complexity, and then used the absolute complexity of sentences as a five-level classification task and propose a new lexical complexity feature based on language learner *corpus* (Pilán & Volodina, 2018). Mohammadi & Khasteh’s (2019) research has achieved automated feature extraction and found the smallest parts needed to evaluate text legibility, effectively utilizing text, which is beneficial for short text legibility assessment (Stajner, Ponzetto & Stuckenschmidt, 2017). Martinc, Pollak & Šikonja (2019) proposed a new readability evaluation method RSRS (ranked sentence readability score) based on a deep neural network language model, which considers two indicators missing in the traditional readability formula: background knowledge and language article linking (Mohammadi & Khasteh, 2019). Experimental results show that, thanks to the trainability of the neural network, the method is able to evaluate the legibility of text according to the context and can be used across languages.

In recent years, the quantitative assessment of the readability of English reading materials has shifted from the measurement of early discourse surface formal features to the measurement of deep-level factors such as latent semantic analysis, semantic association, and presentation (Martinc, Pollak & Šikonja, 2019) of reading comprehension. Among them, Yasuhir, based on the cognitive processing model proposed by Embretson & Wetzel (1987), Howard, Addis & Jing (2007), used discourse characteristics and question characteristics as predictors, and determined each discourse factor and the difficulty factor of the problem factor (Embretson & Wetzel, 1987). By comparing the Flesch-Kincaid grade level formula, the Degrees of Reading Power (DRP) formula, and the Lexile Scores (Lexile Scores) formula, Mcnamara et al. (2014) found that the text difficulty calculated by these formulas was highly correlated, so they set out to develop a Web text analysis tool Coh-Metrix (Ozuru et al., 2008). Coh-Metrix’s theoretical basis is a theoretical framework of multi-faceted factors affecting reading comprehension difficulty, which includes vocabulary, syntax, text structure, referential situational patterns, genre-rhetorical structures, and pragmatic communication between readers and authors dimension. Using tools such as Coh-Metrix, WordSmith, and VocabProfile, Green, Unaldi & Weir (2010) extracted 27 quantitative features of difficulty in academic articles in undergraduate textbooks and IELTS reading questions, and used t-test to compare the two groups of data (Mcnamara et al., 2014). Chen & Ye (2023) proposed an analysis method of fuzzy hierarchy combined with deviation entropy (DE-FAHP) to construct an adaptive English reading comprehension test system ca-MST, which passed the system validity test. The students got satisfactory results in the experiment. put forward an English newspaper reading teaching test model based on deep learning to improve college students’ English proficiency.

In addition to the quantitative evaluation of the readability of English reading materials, the evaluation of students’ English reading ability is also an important link in the process of English teaching and learning, and is an effective measure to improve the quality and effect of English reading learning. In 2017, China released the “China English Proficiency Rating Scale”, which contains the “China English Reading Proficiency Rating Scale”, which provides a scientific basis for describing and evaluating English reading teaching and students’ English reading ability assessment in China’s various academic stages., Uniform standards. Reading ability should be multi-dimensional, that is, reading ability has specific classifications, and it requires various abilities to play a role at the same time (Green, Unaldi & Weir, 2010). In the research on students’ multi-dimensional English reading comprehension assessment, the classification of English reading comprehension is not clearly defined. For example, the Common Core State Standards propose standards for describing reading ability, including four categories ranging from key ideas and details, technique and structure, integration of ideas and knowledge, to scope and discourse difficulty (Wilkins, 2007). Through experiments, Davis measured that students have gone through the processes of understanding the meaning of words, identifying the author’s purpose/attitude/tone, reasoning, answering questions, and grasping the context of the text when reading texts. Guthrie uses factor analysis to extract and identify two major reading ability dimensions for engineers to understand textual meaning and locate key information (Common Core State Standards Initiative, 2020). PIRLS believes that the reading comprehension process is divided into five parts: attention and extraction of clear information, direct inference, interpretation and integration of ideas and information, inspection and evaluation of content, language and discourse elements (Guthrie & Kirsch, 1987).

However, the current research still has some shortcomings. On the one hand, in terms of text readability evaluation methods for English reading materials, the sample size of reading material datasets is small, and a large-scale English text readability evaluation database has not yet been established; on the other hand, legibility based on traditional features The method has been questioned and criticized by many, and the legibility assessment method based on deep learning is less studied. Therefore, it is necessary to use the trainability of neural networks to evaluate the legibility of English text according to the context based on deep learning technology, which is not only more scientific, but also can be used across languages.

## The textcnn based method for evaluating read ability of english textbooks

### The model for evaluating the readability of English textbooks

This study first trains the model using a dataset of English textbook texts with legibility level labels, and then evaluates the legibility level ratings of English textbook texts with unknown labels. Among them, the marked text dataset can be expressed as ${\rm D} = \left\{ {\left( {{{\rm d}_1},{{\rm l}_1}} \right),\left( {{{\rm d}_2},{{\rm l}_2}} \right), \ldots ,\left( {{{\rm d}_{\rm n}},{{\rm l}_{\rm n}}} \right)} \right\}$, ${\rm D}$ represents the marked text set, ${\rm n}$ represents the amount of marked text, ${{\rm d}_{\rm i}}$ represents the i-th marked text in ${\rm D}$, and ${{\rm l}_{\rm i}} \in \left\{ {{{\rm G}_1},{{\rm G}_2}, \ldots ,{{\rm G}_{\rm m}}} \right\}$ is the data. The legibility level corresponding to the concentrated text, where ${\rm m}$ represents the number of legibility levels.

**Figure 1:**
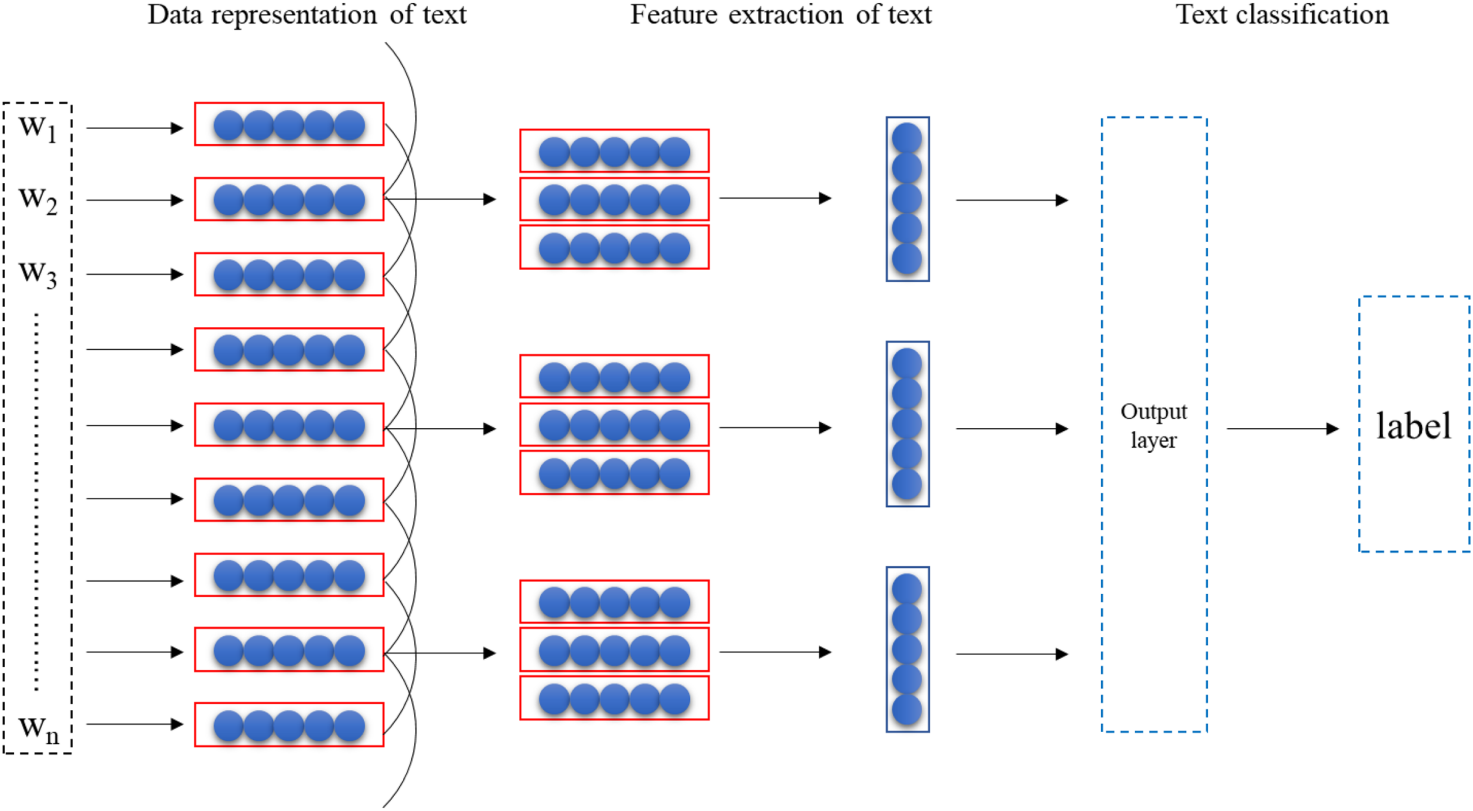
Model structure.

The main task of the text readability evaluation model for English textbooks based on TextCNN is to realize the mapping of text data set ${\rm D} = \left\{ {{{\rm d}_1},{{\rm d}_2}, \ldots ,{{\rm d}_{\rm n}}} \right\}$ to readability level ${{\rm l}_{\rm i}} \in \left\{ {{{\rm G}_1},{{\rm G}_2}, \ldots ,{{\rm G}_{\rm m}}} \right\}$. The model consists of three parts: text data representation, text feature extraction, and text classifier. The model structure is as follows Fig. 1.

#### Data representation of text

The foundational step in leveraging the TextCNN model for the evaluation of text readability in English textbooks is the completion of text representation. This essential process entails employing the Word2vec tool to obtain word vectors, a crucial aspect of data representation. Word2vec exhibits remarkable efficiency in mapping words to numerical vectors, thereby representing words within a multi-dimensional vector space. At the core of Word2vec lies the utilization of deep learning models to systematically train word vectors with specific dimensions.

By completing the text representation, the subsequent stage involves integrating these word vectors into the TextCNN model for a comprehensive evaluation of text readability. The synergy between these components facilitates a nuanced understanding of the intricacies embedded in the textual content of English textbooks. This holistic approach, combining advanced deep learning techniques with effective word vectorization, underscores the sophistication of the methodology employed for evaluating the readability of the text. The integration of Word2vec’s capabilities with the TextCNN model positions the evaluation process to capture nuanced linguistic features, contributing to a robust and insightful analysis of text readability in the context of English textbooks. The degree of similarity between some word vectors can represent the degree of similarity between some words. A sentence of length n (with padding if necessary) is represented by Formula (5):

(5) $${{\rm X}_{{\rm l}:{\rm n}}} = {{\rm x}_1} \oplus {{\rm x}_2} \oplus \ldots \oplus {{\rm x}_{\rm n}}$$

In the provided mathematical expression, the symbol $\oplus$ denotes the concatenation operation. Each row in the represented matrix corresponds to a Word2vec vector of a word, and these vectors are vertically arranged in the order of appearance within the sentence. The input data matrix size is denoted as n × k, where n represents the number of words in the longest sentence within the training data. For the purposes of this study, the value of n is set to 64. The variable k signifies the dimension of the word embeddings. In the current investigation, k is specifically set to 300, reflecting the choice of a 300-dimensional space for the embeddings. This configuration ensures that the input data matrix captures the essential semantic features of the words in the sentence, facilitating the subsequent stages of analysis and modeling within the study.

#### Feature extraction

Convolution kernels of different sizes are used to obtain the relationship between words in different ranges, and the size of one of the convolution kernels is set to be ${\rm k}$. As the convolution kernel slides, for each position ${\rm i}$ in the sequence, there is a window matrix ${{\rm W}_{\rm i}}$ with ${\rm k}$ consecutive words, which is represented as: $\overline {{{\rm W}_{\rm i}}} = \left[ {{{\rm x}_{\rm i}},{{\rm x}_{{\rm i} + 1}},..,{{\rm x}_{{\rm i} + {\rm k} - 1}}} \right]$. The convolution kernel matrix ${\rm M}$ is convolved with the window matrix ${{\rm W}_{\rm i}}$, and then the feature map ${\rm C} \in {{\rm R}^{{\rm L} - {\rm k} + 1}}$ is generated. At the ${\rm i}$-th position, the feature map of the word window vector ${\rm w}$ is calculated as follows:

(6) $${{\rm c}_{\rm i}} = {\rm \sigma }\left( {{\rm w} \oplus {\rm m} + {\rm b}} \right)$$

where b denotes bias, and σ denotes the sigmoid activation function. The subsequent step involves obtaining the feature map through the specified operations.

(7) $${\rm C} = \left[ {{{\rm c}_1},{{\rm c}_2}, \ldots ,{{\rm c}_{{\rm n} - {\rm h} + 1}}} \right]$$

Secondly, use the maximum pooling to act on the result obtained by the convolution calculation, the formula is as follows:

(8) $$ \hat {\rm c} = \max \left\{ {\rm C} \right\}$$

In terms of regularization, dropout is used at the position of the penultimate layer ${\rm Z} = \left[ {\widehat {{{\rm c}_1}},\widehat {{{\rm c}_2}}, \ldots ,\widehat {{{\rm c}_{\rm m}}}} \right]$, and the weight vector is constrained by the ${{\rm l}_2}$ norm, the formula is as follows:

(9) $${\rm y} = {\rm w}\cdot \left( {{\rm z*r}} \right) + {\rm b}$$

when ${\vert \vert {\rm w}\vert\vert_2} > {\rm s}$ after one step of gradient subtraction is performed, ${\rm w}$ is re-decimated to have ${\vert\vert{\rm w}\vert\vert_2} = {\rm s}$ to constrain it with the ${{\rm l}_2}$ norm of the weight vector.

#### Classifiers for text

Finally, in this study, the selected features are assigned to a dense Softmax layer for classification. The classifier section of the English textbook text employs the logistic regression method to construct a multi-class classifier. In this setup, the input vector is the feature vector generated by the CNN. The ultimate representation vector, denoted as v, is derived from the preceding Pooling layer. This vector is then forwarded to the Softmax layer for the classification task. The classification process is mathematically expressed as follows:

(10) $${\rm Softmax}\left( {{{\rm z}_{\rm i}}} \right) = \displaystyle{{{{\rm e}^{{{\rm z}_{\rm i}}}}} \over {\mathop \sum \nolimits_{{\rm c} = 1}^{\rm C} {{\rm e}^{{{\rm Z}_{\rm c}}}}}}$$

In the provided formula, ${{\rm z}_{\rm i}}$ represents the output value of the i-th node, and C represents the number of categories associated with each node. Specifically, C denotes the total number of categories each node is divided into. By employing the Softmax function, the multi-class input values can be transformed into a probability distribution within the range [0, 1], and the probabilities across all categories sum to 1. This conversion ensures that the output from the Softmax layer effectively represents the likelihood of each category, providing a normalized and interpretable output for the multi-class classification task.

In this study, the TextCNN model selects cross entropy as the loss function, and its formula is as follows:

(11) $${\rm L} = \displaystyle{1 \over {\rm N}}\mathop \sum \limits_{\rm i} {{\rm L}_{\rm i}} = \displaystyle{1 \over {\rm N}}\mathop \sum \limits_{\rm i} - \mathop \sum \limits_{{\rm c} = 1}^{\rm M} {{\rm y}_{{\rm ic}}}{\rm log}\left( {{{\rm p}_{{\rm ic}}}} \right).$$

### The introduction of data set

**Figure 2:**
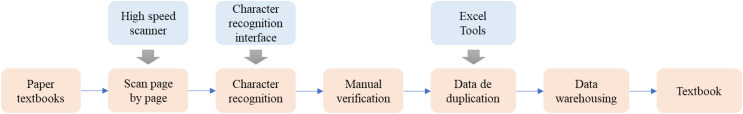
Flow chart of building dataset.

In order to construct the text readability dataset of English textbooks, this study uses three textbooks of different levels of difficulty for college students, “English Extensive Reading 1–3 for College Students”, which is published by Shanghai Foreign Language Education Press. The construction of an English text readability dataset for college students involved a sequential process: scanning textbooks, followed by text recognition, manual verification, data deduplication, and finally, data storage. For a visual representation of this workflow, refer to Fig. 2.

First, use a high-speed scanner to scan the teaching materials to obtain pictures in .jpg format; then, perform text recognition on these pictures to convert them into text format; then, we manually verify the English text, and the researchers correct the incorrectly recognized content, Remove the text whose difficulty does not match the textbook. Finally, use the Excel data deduplication function to realize data deduplication, and finally complete the data storage.

“College Students’ English Extensive Reading” series of books are three English reading textbooks with different levels of difficulty compiled by experts for college students. It is classified as Level 2; Book 3 has the most difficult content and is classified as Level 3 in this study. In order to ensure the sample balance, the number of sentences in each Level is adjusted to be close to 1,000, the number of sentences in Level 1 is 999, the number of sentences in Level 2 is 998, and the number of sentences in Level 3 is 999.

To supplement the English text readability dataset for college students, this study adds “too easy” and “too difficult” sentences, and classifies “too easy” sentences as Level 0 and “too difficult” sentences as Level 4. Among them, the sentence of “too easy” comes from the series of “Oxford English Primary School Textbook” series of Shanghai Education Press, and the sentence of “too difficult” comes from the series of “Comprehensive Course of Advanced College English”.

In order to ensure sample balance, the number of sentences for Level 0 and Level 4 is adjusted to be close to 1,000, the number of sentences for “too easy” is 997, and the number of sentences for “too difficult” is 998. The complete dataset includes the texts of five levels, Level 0, Level 1, Level 2, Level 3 and Level 4, from easy to difficult. The complete dataset is composed as shown in Table 1.

**Table 1:** Number of sentences in the whole dataset.

Level	Number of sentences
Level 0	997
Level 1	999
Level 2	998
Level 3	999
Level 4	998

### The setting of hyper parameters

When training the model on the English textbook text legibility dataset, 20% of the samples were randomly selected as the validation set, and the remaining 80% of the samples were used as the training set. The role of random selection is to offset the differences between individual data and enhance the model effect. In the training phase, the model is configured with specific hyperparameters to optimize its performance. A batch size of 64 is employed, defining the number of samples processed in each iteration. A dropout rate of 0.5 is set, introducing regularization to prevent overfitting. Convolutional kernels with heights of 3, 4, and 5 are utilized, generating a total of 300 Feature Maps with 100 for each kernel height. This convolutional architecture captures diverse contextual information. The learning rate is established at 0.001, influencing the step size taken during optimization to reach the model’s optimal state. These hyperparameter choices collectively contribute to the model’s ability to extract meaningful features from the input data and enhance its generalization performance. The relevant hyperparameter settings are shown in Table 2.

**Table 2:** Setting of hyper parameters.

Hyper parameters	Value
Batch size	64
Dropout	0.5
Kernel size	3, 4, 5
Kernel number	100
Learning rate	0.001

The pseudo-code for the designed model to run is as follows:

**Table:** 

from tensorflow.keras.layers import Input, Embedding, Conv1D, GlobalMaxPooling1D, Dense
from tensorflow.keras.models import Model
from tensorflow.keras.optimizers import Adam
# **hyperparameter**
vocabulary_size = 10,000
max_sequence_length = 100
embedding_size = 100
num_filters = 128
kernel_size = 3
hidden_units = 64
num_classes = 2
input_text = Input(shape=(max_sequence_length,), dtype=‘int32’)
embedding=Embedding(input_dim=vocabulary_size, output_dim=embedding_size, input_length=max_sequence_length)(input_text)
convolution=Conv1D(filters=num_filters,kernel_size=kernel_size,activation=‘relu’)(embedding)
pooling = GlobalMaxPooling1D()(convolution)
dense = Dense(hidden_units, activation=‘relu’)(pooling)
output = Dense(num_classes, activation=‘softmax’)(dense)
model = Model(inputs=input_text, outputs=output)
*model.compile(loss=‘categorical_crossentropy’, optimizer=Adam(), metrics=[‘accuracy’])*

### The analysis of running results

Accuracy is used in this study as an evaluation index. After testing, the evaluation accuracy of the text readability evaluation model for English textbooks based on the TextCNN algorithm proposed in this study is 89.20%. The model is used to evaluate the legibility of sentences in the test set, and the running results are shown in Table 3 below.

**Table 3:** The result of evaluating sentence readability.

Text	Readability assessment
I like the red car.	Level 0
Sue drives to work every day.	Level 1
Now Jessica is working in a hotel in the Florida Keys, one of the warmest places in the United States.	Level 2
One mistake that schools and parents often make is to think that it’s not necessary to teach traffic laws until the child is old enough to drive a car.	Level 3
Primarily, there’s the convenience promised by courses on the Net: you can do the work, as they say, in your pajamas.	Level 4

After obtaining the readability evaluation results of each sentence, we conduct a study on the readability evaluation of English textbook articles. First, the legibility of sentences is evaluated, and the number of sentences with different levels of legibility is counted. Then multiply the number of sentences with legibility of Level 0 by weight 1 to get Score 1, multiply the number of sentences with legibility of Level 1 by weight of 2 to get Score 2, and multiply the number of sentences with legibility of Level 2 by weight of 3 to get Score 3, put The number of sentences with legibility of Level 3 is multiplied by weight 4 to get Score 4, and the number of sentences with legibility of Level 4 is multiplied by weight of 5 to get Score 5. Compare Score 1, Score 2, Score 3, Score 4 and Score 5. If Score 1 is the largest, the legibility of the evaluation article is Level 0; if Score 2 is the largest, the legibility of the evaluation article is Level 1, and so on. The results of evaluating the readability of reading materials using the readability evaluation model are shown in Table 4.

**Table 4:** The result of evaluating passage readability. Bold indicates the highest rated item on each line.

Passage	Score 1	Score 2	Score 3	Score 4	Score 5	Level
1	**6**	2	0	0	0	Level 0
2	0	**34**	9	0	0	Level 1
3	0	2	**21**	8	0	Level 2
4	0	0	9	**32**	0	Level 3
5	0	0	0	16	**60**	Level 4

In this study, the machine learning method is used to match the target text and the graded samples in the dataset, so as to complete the task of grading the difficulty of reading materials. The results show that the results obtained by this model are relatively precise and have certain advantages in evaluating the legibility of English texts in colleges and universities.

## Effectiveness evaluation of text legibility evaluation model

In this study, an experiment was designed to verify the effectiveness of the text readability assessment method based on deep learning by combining the objective results of the subjects’ reading test and the subjective assessment of reading difficulty. Reading level, develop reading interest, enhance reading confidence. The subjects of the experiment were freshmen in two classes of computer science in a university in China. The computer class 1 served as the experimental group with 44 students, and the computer class 2 served as the control group with 41 students. The experimental group applied the text readability evaluation method based on deep learning, while the control group did not. We collected the reading test data and the reading material difficulty assessment results of the experimental group and the control group, and then used SPSS22.0 statistical software for data analysis.

This research collects the real reading comprehension questions in the CET-4 test of Chinese college students, and finds the articles that meet the difficulty of Level 0–Level 4 as the reading test data, corresponding to Passage 1, Passage 2, Passage 3, Passage 4 and Passage 5. After reading each passage, students need to complete five multiple-choice questions and choose one correct option from the options.

**Figure 3:**
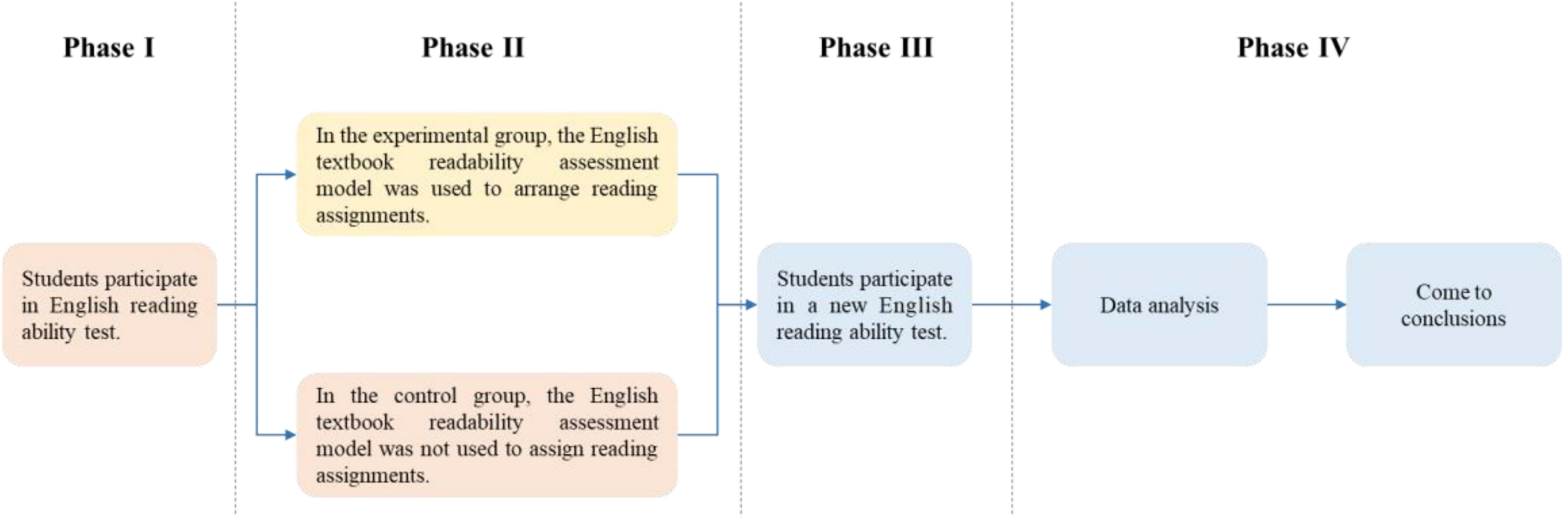
Flow chart of experiment.

In this experiment, computer class 1 and computer class 2 were selected for a 16-week teaching experiment. The experiment divided the 16-week experimental teaching into four stages, as shown in Fig. 3.

The first stage (week 1): use the same reading test questions to test the students in the experimental group and the control group to determine whether there is a significant difference in the perception of the difficulty of the text and the reading ability of the subjects.

The second stage (weeks 2–15): Select reading materials, and use the TextCNN-based English textbook readability evaluation model to classify the reading materials into Level 0–Level 4. In the experimental group, students were assigned reading materials one Level higher than their current Level, while the control group did not assign reading materials according to the students’ current Level.

The third stage (week 16): A new reading ability test was conducted for students in two classes, and then a questionnaire survey was conducted for students in the experimental group to conduct a subjective evaluation of the role of the TextCNN-based English textbook readability evaluation model.

The fourth stage: use SPSS22.0 tools to analyze the collected data and draw conclusions.

## Result analysis and discussion

The English reading comprehension test data of the students in the two classes were collected, and the accuracy, mean and standard deviation were calculated. The statistical results show that there is no significant difference in the English proficiency of computer class 1 and class 2 students from the three evaluation results of accuracy, mean and standard deviation. As shown in Table 5.

**Table 5:** Answer data of computer class 1 and class 2 before experiment.

	Accuracy		Average value		Standard deviation	
Passage	Computer class 1	Computer class 2	Computer class 1	Computer class 2	Computer class 1	Computer class 2
1	61.36%	60.98%	35.73%	35.22%	12.15	12.23
2	48.64%	47.80%				
3	33.64%	32.68%				
4	21.36%	20.98%				
5	13.64%	13.66%				

According to the English reading comprehension test scores, students’ English reading ability is divided into different levels: 0–20 is divided into Level 0, 21–40 is divided into Level 1, 41–60 is divided into Level 2, 61–80 is divided into Level 3, 81–100 divided into Level 4. Assuming that the student’s current Level is i, the Level of reading materials assigned to this student in this study is i + 1.

The main purpose of the subjective perception of reading difficulty part is to examine students’ subjective perception of the difficulty of the text. Ask students to answer whether they can read the text according to their own feelings, and rank the five texts according to difficulty. The evaluation method is designed according to the Likert scale. The English reading materials are scored according to the standards of complete comprehension, basic comprehension, partial comprehension, basic comprehension and complete comprehension, with scores of 5, 4, 3, 2 and 1 respectively.

Collect the subjective evaluation data of students’ reading difficulty, count the number of people who can fully understand or basically understand each Passage, and calculate the proportion. The reading situation is shown in Table 6.

**Table 6:** Students’ understanding of the passages in computer class 1 and class 2.

	Computer class 1		Computer class 2	
Passage	Fully or basically comprehend	The proportion	Fully or basically comprehend	The proportion
1	35	79.55%	30	73.17%
2	29	65.91%	26	63.41%
3	23	51.11%	21	51.22%
4	18	40.91%	17	41.46%
5	5	11.36%	2	4.88%

In class 1 and class 2, the number of students’ understanding of reading materials Passage 1 to Passage 5 is decreasing, and the results of students’ subjective assessment of reading difficulty are consistent with the objective results of reading tests and the results of the evaluation model of English textbook readability.

We count the number of each message that is ranked as the easiest, easier, medium, harder, and hardest. The ranking of students in computer class 1 is shown in Table 7, and the ranking of students in computer class 1 is shown in Table 8.

**Table 7:** Students’ ranking of the difficulty of passages in computer class 1. Bold indicates the highest rated item on each line.

Passage	Easiest	Easier	Medium	Difficult	Hardest	Total	Level
1	**44**	2	0	0	0	44	Level 0
2	0	**29**	9	6	0	44	Level 1
3	0	9	**23**	12	0	44	Level 2
4	0	6	12	**25**	1	44	Level 3
5	0	0	0	1	**43**	44	Level 4

**Table 8:** Students’ ranking of the difficulty of passages in computer class 2. Bold indicates the highest rated item on each line.

Passage	Easiest	Easier	Medium	Difficult	Hardest	Total	Level
1	**41**	0	0	0	0	41	Level 0
2	0	**22**	9	10	0	41	Level 1
3	0	9	**21**	11	0	41	Level 2
4	0	10	11	**17**	3	41	Level 3
5	0	0	0	3	**38**	41	Level 4

In 85 subjects, all of them gave difficulty ranking. In class 1 and class 2 of Computer Science, most students think that Passage 1 (with a readability level of Level 0) is the easiest, most students think that Passage 2 (with a readability level of Level 1) is the easier, most students think that Passage 3 (with a readability level of Level 2) is the middle, most students think that Passage 4 (with a readability level of Level 3) is the harder, and most students think that Passage 5 (with a readability level of Too difficulty) is the hardest. It can be seen that the subjective feelings of these students are consistent with the results of the text readability assessment method, indicating the effectiveness of this model, which can be used to improve the English reading ability of students at different levels in the process of learning English.

In the 16th week, we conducted a new reading ability test for the two classes to find out whether the readability assessment model can promote students’ English reading ability. Table 9 shows the answer accuracy data of computer class 1 and class 2.

**Table 9:** Answer data of computer class 1 and computer class 2 after experiment.

	Accuracy	
Passage	Computer class 1	Computer class 2
1	77.27%	62.44%
2	60.45%	47.32%
3	40.00%	36.10%
4	26.36%	22.93%
5	14.64%	11.15%

In class 1 and class 2, the number of correct answers in Passage 1 to Passage 5 is decreasing, and the results of reading test are consistent with the results of text readability evaluation method, which again proves that the text readability evaluation method based on deep learning is scientific. Moreover, there is a significant difference between class 1 and class 2 in English reading level. With the intervention of the readability evaluation model, the progress of the students in class 1 is better than that in class 2. It can be seen that the application of the readability evaluation model of reading materials can improve the students’ English reading level, which proves that the application of the text readability evaluation method based on in-depth learning can improve the students’ English reading level, and proves the effectiveness of the model.

According to the above analysis of experimental results of the experimental group and the control group, this study uses convolutional neural network to evaluate the readability of English reading materials, and uses this model to provide English reading materials of appropriate difficulty for students according to their English reading ability, so as to improve the English learning effect. This article constructs TextCNN text readability evaluation model and conducts experimental teaching on self-built data set. The final assessment accuracy rate reaches 90%, and the reading level and reading interest of students in the experimental group are significantly improved, which proves that the text readability method based on deep learning is scientific and effective. However, this method is based on self-built data set, not based on public data set and lack of comparison with other models, so the model in this article lacks generalization ability.

## Conclusion

This study constructs a text readability evaluation model based on TextCNN deep learning algorithm, and applies this model to college English teaching. SPSS22.0 statistical software is used to analyze the collected experimental data, which verifies the scientificity and effectiveness of this method in improving students’ English reading ability. The experiment was carried out in different ways when two classes of computer major in a university assigned reading assignments after class. The experimental group applied the text readability evaluation method based on deep learning, while the control group did not.

For the reading material Passage 1 with the text readability evaluation result of Level 0, the number of correct answers is the largest, and for the reading material Passage 5 with the text readability evaluation result of Level 4, the number of correct answers is the smallest. This shows that the results of the objective English reading test are consistent with the text readability assessment results, and proves that the text readability assessment method based on deep learning is scientific. For the reading material Passage 1 with the text readability evaluation result of Level 0, the subjects subjectively read the most; for the reading material Passage 5 with the text readability evaluation result of Level 4, the subjects subjectively thought it was the most difficult. It shows that the subjective assessment results of the subjects are consistent with the text readability assessment results, which further proves the scientificity of the text readability assessment method based on deep learning. There is no significant difference in the reading test scores between the experimental group and the control group before the experiment. However, under the effect of the text readability evaluation method based on depth learning, there is a significant difference in the reading scores between the experimental group and the control group at the 16th week, which indicates that the application of the text readability evaluation method based on depth learning can significantly improve the reading level of college students, and proves the effectiveness of the text readability evaluation method based on depth learning.

Although the model for evaluating the readability of college students’ English texts based on deep learning proposed in this study has made some research achievements, it still has the following shortcomings: First of all, this study only collects English texts from the extensive reading of college students’ English 1–3, Oxford English primary school textbooks and comprehensive college advanced English courses, and the capacity of the data set needs to be further expanded in the future; Secondly, the amount of sample data in this research experiment is small. The research object selects two classes of computer major in a university, a total of 85 students. The number of classes and students is small, and the universality of the results is limited. In the future, this article can obtain a control group experiment through more data of class and college students’ learning. Besides text readability, English learning can also be strengthened from other perspectives.

## Supplemental Information

10.7717/peerj-cs.1895/supp-1 Supplemental Information 1 Codes.

## Additional Information and Declarations

### Data Availability

The following information was supplied regarding data availability: The codes are available in the Supplemental File. The data is available at Zenodo: Le Foll, E. (2021). Bibliographic metadata of the Textbook English *Corpus* (TEC) (v. 1.1) [Data set]. Zenodo. https://doi.org/10.5281/zenodo.4922819.
